# Practical considerations for optimising homologous recombination repair mutation testing in patients with metastatic prostate cancer

**DOI:** 10.1002/cjp2.203

**Published:** 2021-02-25

**Authors:** David Gonzalez, Joaquin Mateo, Albrecht Stenzinger, Federico Rojo, Michelle Shiller, Alexander W Wyatt, Frédérique Penault‐Llorca, Leonard G Gomella, Ros Eeles, Anders Bjartell

**Affiliations:** ^1^ Patrick G Johnston Centre for Cancer Research Queen's University Belfast UK; ^2^ Vall d'Hebron Institute of Oncology (VHIO) Vall d'Hebron University Hospital Barcelona Spain; ^3^ Institute of Pathology University Hospital Heidelberg Heidelberg Germany; ^4^ Department of Pathology IIS‐Hospital Universitario Fundación Jiménez Díaz‐CIBERONC Madrid Spain; ^5^ Department of Pathology Baylor University Medical Center Dallas TX USA; ^6^ Vancouver Prostate Centre, Department of Urologic Sciences University of British Columbia Vancouver BC Canada; ^7^ Centre Jean Perrin Université Clermont Auvergne, INSERM, U1240 Imagerie Moléculaire et Stratégies Théranostiques Clermont Ferrand France; ^8^ Department of Urology, Sidney Kimmel Cancer Center Thomas Jefferson University Philadelphia PA USA; ^9^ Division of Genetics and Epidemiology The Institute of Cancer Research and The Royal Marsden NHS Foundation Trust London UK; ^10^ Division of Urological Cancers, Department of Translational Medicine Lund University Lund Sweden

**Keywords:** metastatic prostate cancer, molecular diagnostics, homologous recombination repair, poly(ADP‐ribose) polymerase inhibitors, mCRPC

## Abstract

Analysis of the genomic landscape of prostate cancer has identified different molecular subgroups with relevance for novel or existing targeted therapies. The recent approvals of the poly(ADP‐ribose) polymerase (PARP) inhibitors olaparib and rucaparib in the metastatic castration‐resistant prostate cancer (mCRPC) setting signal the need to embed molecular diagnostics in the clinical pathway of patients with mCRPC to identify those who can benefit from targeted therapies. Best practice guidelines in overall biospecimen collection and processing for molecular analysis are widely available for several tumour types. However, there is no standard protocol for molecular diagnostic testing in prostate cancer. Here, we provide a series of recommendations on specimen handling, sample pre‐analytics, laboratory workflow, and testing pathways to maximise the success rates for clinical genomic analysis in prostate cancer. Early involvement of a multidisciplinary team of pathologists, urologists, oncologists, radiologists, nurses, molecular scientists, and laboratory staff is key to enable optimal workflow for specimen selection and preservation at the time of diagnosis so that samples are available for molecular analysis when required. Given the improved outcome of patients with mCRPC and homologous recombination repair gene alterations who have been treated with PARP inhibitors, there is an urgent need to incorporate high‐quality genomic testing in the routine clinical pathway of these patients.

## Introduction

**Figure 1:**
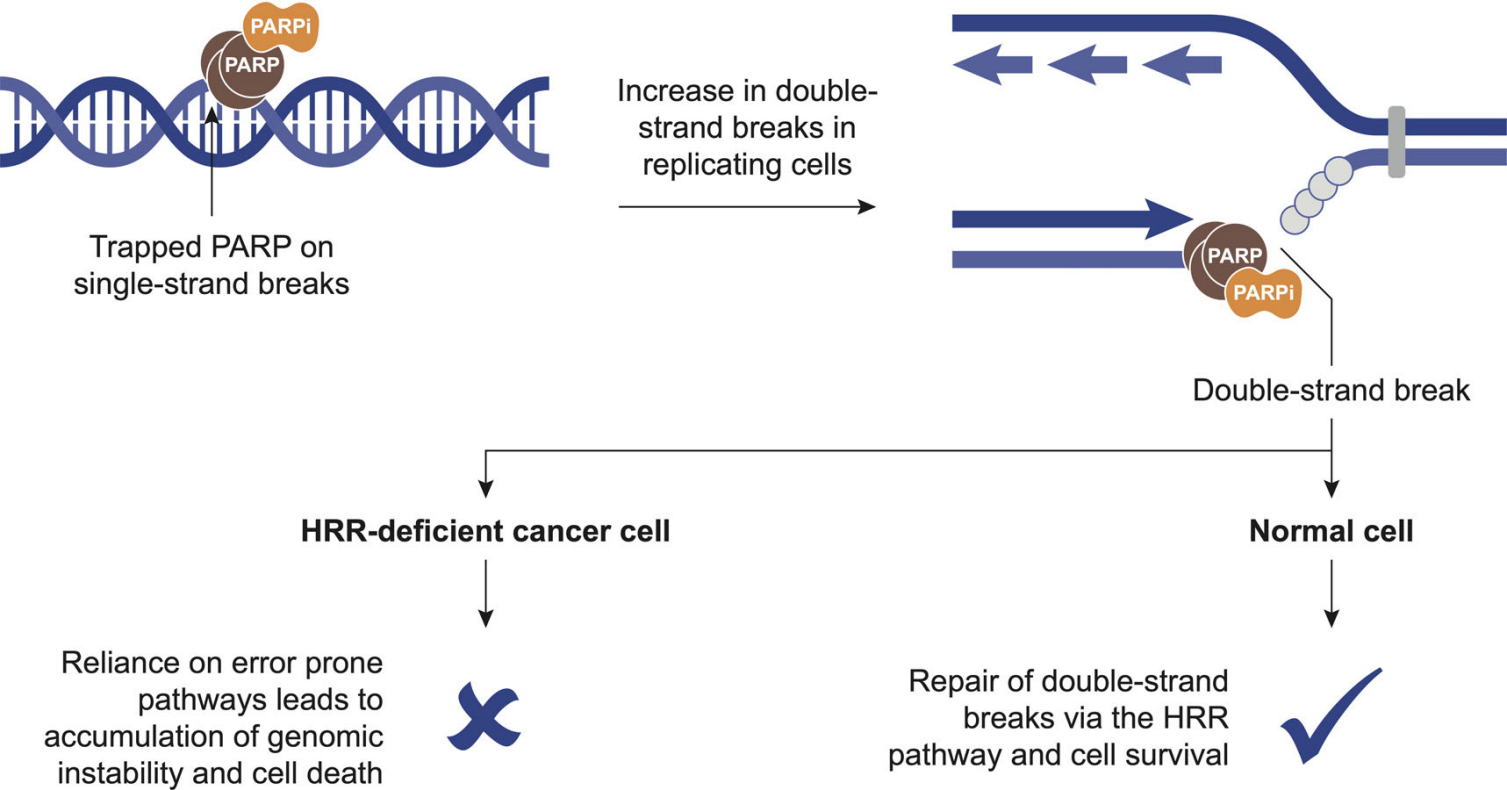
Schematic mechanism of PARP inhibition. PARP inhibitors trap PARP at sites of single‐strand breaks, leading to generation of double‐strand breaks and cell death in cancer cells with deficiency in HRR. PARPi, PARP inhibitor. Adapted from O'Connor [3].

Prostate cancer is a heterogeneous disease with a variable prognosis depending on the stage at diagnosis, as well as other clinical and biological factors. Most patients are diagnosed with curable disease, but approximately 15% of patients will present with, or eventually develop, metastatic disease and resistance to androgen‐based therapies; for this group of patients, there has been a significant improvement in treatment approaches with the development of targeted agents [1]. One novel class of targeted agents, poly(ADP‐ribose) polymerase (PARP) inhibitors, is beneficial for selected patients with metastatic castration‐resistant prostate cancer (mCRPC). PARP enzymes have a key role in DNA repair, but when PARP inhibitors catalytically inhibit PARylation and physically ‘trap’ PARP on DNA at sites of single‐strand breaks, they prevent DNA repair via the base‐excision repair pathway [2]. This leads to the generation of double‐strand breaks which cannot be efficiently repaired in tumour cells that have defects in the homologous recombination repair (HRR) pathway, causing accumulation of DNA damage and tumour cell death (Figure 1) [3, 4]. This mechanism of action is known as synthetic lethality, where deleterious (i.e. pathogenic or likely pathogenic) HRR gene alterations can confer sensitivity to PARP inhibition, and has been demonstrated in prostate cancer, as well as ovarian, pancreatic, and breast cancer [5, 6, 7].

Commonly reported genomic alterations in mCRPC include mutations and copy number alterations in genes such as *TP53*, *AR*, *RB1*, *PTEN*, and those involved in repairing DNA damage, predominantly those with a role in HRR [8]. Table 1 details HRR genes where genomic alterations have been reported across different tumour types in the literature. Recent studies have shown that approximately 25% of patients with mCRPC harbour deleterious alterations in genes directly or indirectly involved in HRR that may act as biomarkers of response to PARP inhibitors (Table 2) [9, 10, 16, 17]. With the introduction of targeted agents into clinical practice, molecular diagnostic profiling is required to identify patients who may benefit from these therapies. One commonly used method for HRR assessment in mCRPC is the sequencing of DNA extracted from tumour tissue specimens as it captures patients with both germline and somatic alterations. If necessary, subsequent germline testing can be used to resolve whether an alteration is germline or somatic as tumour tissue tests cannot distinguish between these. Tumour material for testing is obtained from archival tissue biopsy specimens. Given that the majority of HRR alterations in prostate cancer are either germline or appear to occur early in the disease and prior to metastatic spread [18, 19, 20, 21], evaluation of dominant tumour focus (high volume/grade) in archival diagnostic specimens is appropriate for molecular diagnostics even after mCRPC progression [22]. Indeed, the molecular selection of patients with metastatic disease based on testing of primary tumours has been the main strategy for patient enrolment in the pivotal PARP inhibitor trials for patients with mCRPC [9, 11].

**Table 1:** Altered HRR genes identified in various tumour types.

Tumour type	HRR genes reported
Prostate [9, 10, 11,16]	*ATM*, *ATR*, *BARD1*, *BRCA1*, *BRCA2*, *BRIP1*, *CDK12*, *CHEK1*, *CHEK2*, *FAM175A*, *FANCA*, *FANCL*, *GEN1*, *MRE11A*, *MSH2*, *MSH6*, *PALB2*, *PPP2R2A*, *NBN*, *RAD51*, *RAD51B*, *RAD51C*, *RAD51D*, *RAD54L*
Breast [12]	*ARID1A*, *ATM*, *ATRX*, *BAP1*, *BARD1*, *BLM*, *BRCA1*, *BRCA2*, *BRIP1*, *CHEK1*, *CHEK2*, *FANCA*, *FANCC*, *FANCD2*, *FANCE*, *FANCF*, *FANCG*, *FANCL*, *KMT2D*, *MRE11A*, *NBN*, *PALB2*, *RAD50*, *RAD51*, *RAD51B*, *WRN*
Ovarian [13, 14]	*BRCA1*, *BRCA2*, *ATM*, *ATR*, *BARD1*, *BLM*, *BRIP1*, *CHEK2*, *MRE11A*, *NBN*, *PALB2*, *RAD51C*, *RAD51D*, *RBBP8*, *SLX4*, *XRCC2*
Multiple [15]	*ARID1A*, *ATM*, *ATRX*, *BAP1*, *BARD1*, *BLM*, *BRCA1*, *BRCA2*, *BRIP1*, *CHEK1*, *CHEK2*, *FANCA*, *FANCC*, *FANCD2*, *FANCE*, *FANCF*, *FANCG*, *FANCL*, *MRE11A*, *NBN*, *PALB2*, *RAD50*, *RAD51*, *RAD51B*, *WRN*

**Table 2:** Most prevalent HRR alterations in patients with mCRPC.

Study	Patients	HRR alteration	Prevalence (%)	
			Germline only	Tissue testing*
Robinson *et al* [16]	150 with mCRPC underwent testing of metastatic biopsies	*ATM*	NR	7.3
		*BRCA2*		13.3
		*BRCA1*		0.7
		*CDK12*		4.7
		*MLH1*		0.7
		*MSH2*		2.0
Abida *et al* [17]	451 patients with mCRPC provided 504 tissue samples for tumour testing, and 221 underwent germline testing. Of these, 27% had germline or somatic mutations	*ATM*	2.3	6.8
		*BRCA2*	8.6	16.3
		*BRCA1*	0.9	1.8
		*CHEK2*	4.1	5.0
Pritchard *et al* [10]	692 with mCRPC underwent germline testing, of whom 82 (11.8%) had pathogenic germline HRR alterations^†^	*ATM*	1.6	NR
		*BRCA2*	5.3	
		*BRCA1*	0.9	
		*CHEK2*	1.9	
		*GEN1*	0.5	
		*PALB2*	0.4	
		*RAD51D*	0.4	
de Bono *et al* (PROfound) [18]	4047 with mCRPC underwent testing; 2792 (69%) had a successful test. Of these, 778 (27.9%) had either germline or somatic alterations^‡^	*ATM*	NR	5.9
		*BRCA2*		8.7
		*BRCA1*		1.0
		*BRIP1*		0.4
		*CDK12*		6.3
		*CHEK2*		1.2
		*PALB2*		0.3
		*PPP2R2A*		1.0
		*RAD51B*		0.3

NR, not reported.

* Tumour tissue testing detects both germline and somatic alterations, but germline testing is needed to confirm if alterations are of germline origin.

^†^ Other alterations identified from germline testing in <0.3% of patients include *BRIP1*, *FAM175A*, *MRE11A*, *MSH2*, *MSH6*, *NBN*, *PMS2*, and *RAD51C*.

^‡^ Other alterations identified from tumour testing in <0.3% of patients include *BARD1*, *CHEK1*, *RAD51D*, and *RAD54L* (one patient had an *FANCL* alteration, but none had *RAD51C* alterations).

## Opportunities and challenges in mCRPC

Real‐world data on the testing success of prostate tumour samples are limited as clinical next‐generation sequencing (NGS) has only recently been implemented for this tumour type outside of the context of clinical trials. However, in clinical trials to date, attrition rates of approximately 30–40% have been reported for strategies relying on tumour tissue testing in patients with mCRPC [9, 17, 23, 24]. Consequently, there is an urgent need to significantly improve testing approaches. The main reasons for test failures appear to be: (1) the limited amount of tumour tissue collected during diagnostic biopsies, (2) exhaustion of diagnostic material during the histological diagnosis, (3) insufficient tumour content for genomic analysis, and (4) suboptimal DNA yield/quality due to DNA degradation during fixation and/or storage of diagnostic material [9, 17, 25].

The aim of this review is to provide practical considerations and recommendations for molecular diagnostic testing of specimens collected from patients with mCRPC in clinical practice with a focus on optimizing the success rates for multigene NGS assays. For the purpose of this manuscript, HRR genes refer generically to *BRCA1* and *BRCA2*, at a minimum, and to a larger variety of genes known to be involved directly or indirectly in the HRR pathway (Tables 1 and 2).

## Overview of PARP inhibitor studies in mCRPC

Several PARP inhibitors have been evaluated in studies of patients with mCRPC, many of which have included prospective selection for HRR alterations prior to treatment [7, 11, 24, 26, 27, 28, 29, 30]. The phase II PARP inhibitor monotherapy studies TOPARP‐B (olaparib), TRITON2 (rucaparib), TALAPRO‐1 (talazoparib), and GALAHAD (niraparib) identified responses in patients with germline or somatic HRR alterations, although higher response rates and longer duration of responses were generally observed in those with *BRCA1* and *BRCA2* alterations (Table 3) [24, 27, 28, 29].

**Table 3:** Phase II and III studies of PARP inhibitors in mCRPC.

Study	mCRPC population	PARP inhibitor (monotherapy)	Sample type for testing; assay	Gene alterations evaluated	Types of gene alterations evaluated	Primary end point
**Phase II**						
TOPARP‐B [24]	Of 711 patients screened, 592 had evaluable samples. Of these, 161 (27%) had DDR gene aberrations; of these, 98 were randomised to treatment (49 to each olaparib group)	Olaparib: 300 mg BID or 400 mg BID	*De novo* or archival FFPE primary or metastatic; FoundationOne CDx	*BRCA2*, *BRCA1*, *ATM*, *CDK12*, *PALB2*, *ARID1A*, *ATRX*, *CHEK1*, *CHEK2*, *FANCA*, *FANCF*, *FANCG*, *FANCI*, *FANCM*, *MSH2*, *NBN*, *RAD50*, *WRN*	Mono‐ or biallelic alterations; homozygous deletion or deleterious mutations	**Confirmed composite response** * *BRCA1/BRCA2*: (25/30) 83.3% (95% CI: 65.3–94.4) *ATM*: (7/19) 36.8% (95% CI: 16.3–61.6) *CDK12*: (5/20) 25.0% (95% CI: 8.7–49.1) *PALB2*: (4/7) 57.1% (95% CI: 18.4–90.1) Other: (4/20) 20.0% (95% CI: 5.7–43.7)
TRITON‐2 [27]	190 patients with HRR mutations previously treated with abiraterone, enzalutamide, docetaxel, or cabazitaxel	Rucaparib: 600 mg BID	*De novo* or archival FFPE samples; FoundationOne CDx Plasma; FoundationOne Liquid CDx	*BRCA1*, *BRCA2*, *ATM*, *CDK12*, *CHEK2*, *BARD1* (others include *BRIP1*, *FANCA*, *NBN*, *PALB2*, *RAD51*, *RAD51B*, *RAD51C*, *RAD51D*, *RAD54L*)	Mono‐ or biallelic alterations; deleterious mutations	**ORR (RECIST/PCWG3 criteria)** *BRCA1/BRCA2*: (25/57) 43.9% (95% CI: 30.7–57.6) *ATM*: (2/21) 9.5% (95% CI: 1.2–30.4) *CDK12*: (0/9) 0% (95% CI: 0.0–33.6) *CHEK2*: (0/5) 0% (95% CI: 0.0–52.2) Others: (5/13) 38.5% (95% CI: 13.9–68.4)
TALAPRO‐1 [28]	81 patients with DDR mutations. Patients had progressed on taxane or AR signalling inhibitor	Talazoparib: 1 mg OD	*De novo* or archival tumour tissue; FoundationOne CDx	*ATM*, *ATR*, *BRCA1*, *BRCA2*, *CHEK2*, *FANCA*, *MLH1*, *MRE11A*, *NBN*, *PALB2*, *RAD51C*	NA	**ORR (RECIST v1.1)** Overall: 25.6% (95% CI: 13.5–41.2) *BRCA1/BRCA2*: 50% (95% CI: 27.2–72.8) *ATM*: 7.1% (95% CI: 0.2–33.9)
GALAHAD [29]	Of 223 patients screened, 165 with mCRPC and DNA repair defects (mono‐ or biallelic BRCA and non‐BRCA) were enrolled and 81 with biallelic mutations (46 *BRCA* and 35 non‐*BRCA*) comprised the primary population. Patients had progressed on taxane or AR signalling inhibitor	Niraparib: 300 mg OD	Biallelic alteration blood or tissue assay, and germline pathogenic *BRCA1/BRCA2* by any test	*BRCA1*, *BRCA2*, *ATM*, *FANCA*, *PALB2*, *CHEK2*, *BRIP1*, *HDAC2*	Biallelic alterations (including homozygous deletions)	**ORR (RECIST 1.1 and PCWG3 criteria)** Biallelic^†^ *BRCA1/BRCA2*: (12/29) 41% (95% CI: 23.5–61.1) Secondary end point: biallelic non‐BRCA: (2/22) 9% (95% CI: 1.1–29.2)
**Phase III**						
PROfound [9, 31]	Of 4047 patients with mCRPC who underwent testing, 2792 (69%) had a successful test. Of these, 778 (27.9%) had either germline or somatic alterations	Olaparib: 300 mg BID Control: either enzalutamide (160 mg OD) or abiraterone (1000 mg OD plus prednisone [5 mg BID])	*De novo* or archival FFPE primary or metastatic; investigational clinical trial assay based on FoundationOne CDx	Cohort A: *BRCA2*, *BRCA1*, *ATM* Cohort B: *BARD1*, *BRIP1*, *CDK12*, *CHEK1*, *CHEK2*, *FANCL*, *PALB2*, *PPP2R2A*, *RAD51B*, *RAD51C*, *RAD51D*, *RAD54L*	Mono‐ or biallelic alterations; homozygous deletion or deleterious mutations	**Imaging‐based PFS (cohort A)** 7.4 months for olaparib versus 3.6 months for control; hazard ratio 0.34 (95% CI: 0.25–0.47; *p* < 0.001) **Secondary end points:** **Imaging‐based PFS (overall population)** 5.8 months for olaparib versus 3.5 months for control; hazard ratio 0.49 (95% CI: 0.38–0.63; *p* < 0.001) **Overall survival (cohort A)** Median duration was 19.1 months for olaparib and 14.7 months for control; hazard ratio 0.69 (95% CI: 0.50–0.97; *p* = 0.02) **Overall survival (overall population)** Median duration was 17.3 months for olaparib and 14.0 months for control; hazard ratio 0.79 (95% CI: 0.61–1.03)

95% CI, 95% confidence interval; AR, androgen receptor; BID, twice daily; DDR, DNA damage repair; FFPE, formalin‐fixed and paraffin‐embedded; NA, not available; OD, once daily; ORR, objective response rate; PCWG3, Prostate Cancer Working Group 3; PFS, progression‐free survival; RECIST, Response Evaluation Criteria in Solid Tumors.

^*^ Defined as radiological objective response (as assessed by RECIST), a decrease in prostate‐specific antigen of 50% or more from baseline, or conversion of circulating tumour cell count (from ≥5 cells per 7.5 ml blood at baseline to <5 cells per 7.5 ml blood).

^†^ Homozygous/compound heterozygous mutation or mutation in one allele with loss of the other allele.

The PROfound study was the first randomised phase III study demonstrating the efficacy of a PARP inhibitor, olaparib, in patients with mCRPC [9]. In PROfound, treatment with olaparib was associated with significantly longer progression‐free survival and overall survival than enzalutamide or abiraterone (control) in patients who had at least one alteration in *BRCA1*, *BRCA2*, or *ATM* (cohort A) and had disease progression while receiving enzalutamide or abiraterone (see Table 3 for details) [9, 31]. Based on the findings of the PROfound trial, the Food and Drug Administration (FDA) approved olaparib for adult patients with deleterious or suspected deleterious germline or somatic HRR gene‐mutated mCRPC who have progressed following prior treatment with enzalutamide or abiraterone [32]. In addition, the European Medicines Agency approved olaparib as monotherapy for the treatment of adult patients with mCRPC and *BRCA1* or *BRCA2* mutations (germline and/or somatic) whose disease progressed following prior therapy that included a next‐generation hormonal agent [33]. Rucaparib was also approved by the FDA for patients with deleterious *BRCA1* or *BRCA2* mutation (germline and/or somatic)‐associated mCRPC who have been treated with androgen receptor‐directed therapy and a taxane‐based chemotherapy based on the tumour testing findings of the TRITON2 study [34]. A phase III study (TRITON3) of rucaparib in patients with mCRPC and a deleterious germline or somatic *BRCA1*, *BRCA2*, or *ATM* mutation is ongoing [35]. Breakthrough therapy designation has also been granted by the FDA for niraparib based on the findings of the GALAHAD study [29], and other approvals are anticipated. Beyond differences in the PARP inhibitors being evaluated, these trials differed in the patient selection strategy and also used different assays, including tissue and liquid biopsy‐based testing of slightly different panels of HRR genes. However, these studies support the importance of genomic profiling and the implementation of molecular analysis in the clinical pathway.

## Current tumour testing guidelines for prostate cancer

The US National Comprehensive Cancer Network (NCCN) guidelines were updated in 2019 to recommend tumour testing for HRR gene alterations and consider microsatellite instability (MSI)/mismatch repair testing in all patients with regional or metastatic prostate cancer [36, 37]. This information may be used for genetic counselling, eligibility for PARP inhibitor treatment, or enrolment in clinical trials. If pathogenic or likely pathogenic alterations in *BRCA1*, *BRCA2*, *ATM*, *PALB2*, and *CHEK2* are found, and/or there is a strong family history of cancer, then patients should be referred for genetic counselling and confirmatory germline testing. The Advanced Prostate Cancer Consensus Conference held in 2019 supported consideration of *BRCA1* and *BRCA2* testing in screening, management, and informing prognosis/treatment, with germline testing recommended in patients with a tumour *BRCA1*, *BRCA2*, or *ATM* mutation [38]. Similar recommendations for germline testing were published by the 2019 Philadelphia International Prostate Cancer Consensus that supported the use of prostate cancer gene‐testing panels [39]. The American Urological Association/American Society for Radiation Oncology/Society of Urologic Oncology (AUA/ASTRO/SUO) guidelines published in June 2020 state that patients with mCRPC should be offered tumour and/or germline HRR gene testing and MSI status [40]. More recently, the European Society of Medical Oncology (ESMO) clinical practice guidelines for diagnosis, treatment, and follow up of prostate cancer were updated to provide guidance for precision medicine [41]. The ESMO Precision Medicine Working Group recommends that multigene NGS panel testing replace single‐gene assays and be considered for patients with metastatic prostate cancer, and those with pathogenic or likely pathogenic mutations in cancer‐risk genes should be referred for genetic counselling and germline testing for *BRCA1/BRCA2* and other HRR alterations [42]. While there may be variations in testing recommendations, access to testing, and reimbursement issues between countries, analyses of somatic and germline *BRCA1* and *BRCA2* alterations are likely to become the minimum requirement in many countries for patients with mCRPC.

## Integrating genomic testing in the patient pathway

Tumour tissue collection in prostate cancer is predominantly driven by diagnostic need, particularly as pathological tumour typing is directly related to clinical management and, ultimately, patient outcome. In current practice, tissue‐based molecular diagnostic testing (that identifies mutations that could be of somatic or germline origin) is most likely to be requested at the point when a patient develops metastatic disease, aligned to access to biomarker‐targeted therapies. However, for patients with a strong family history of cancer, germline screening for cancer predisposition genes may be requested even when only local/regional disease is present. Understanding regional differences in diagnostic policies and capabilities will be important to provide appropriate guidance for the successful introduction of molecular diagnostic testing in the community setting.

## Prostate tissue sampling in practice

**Figure 2:**
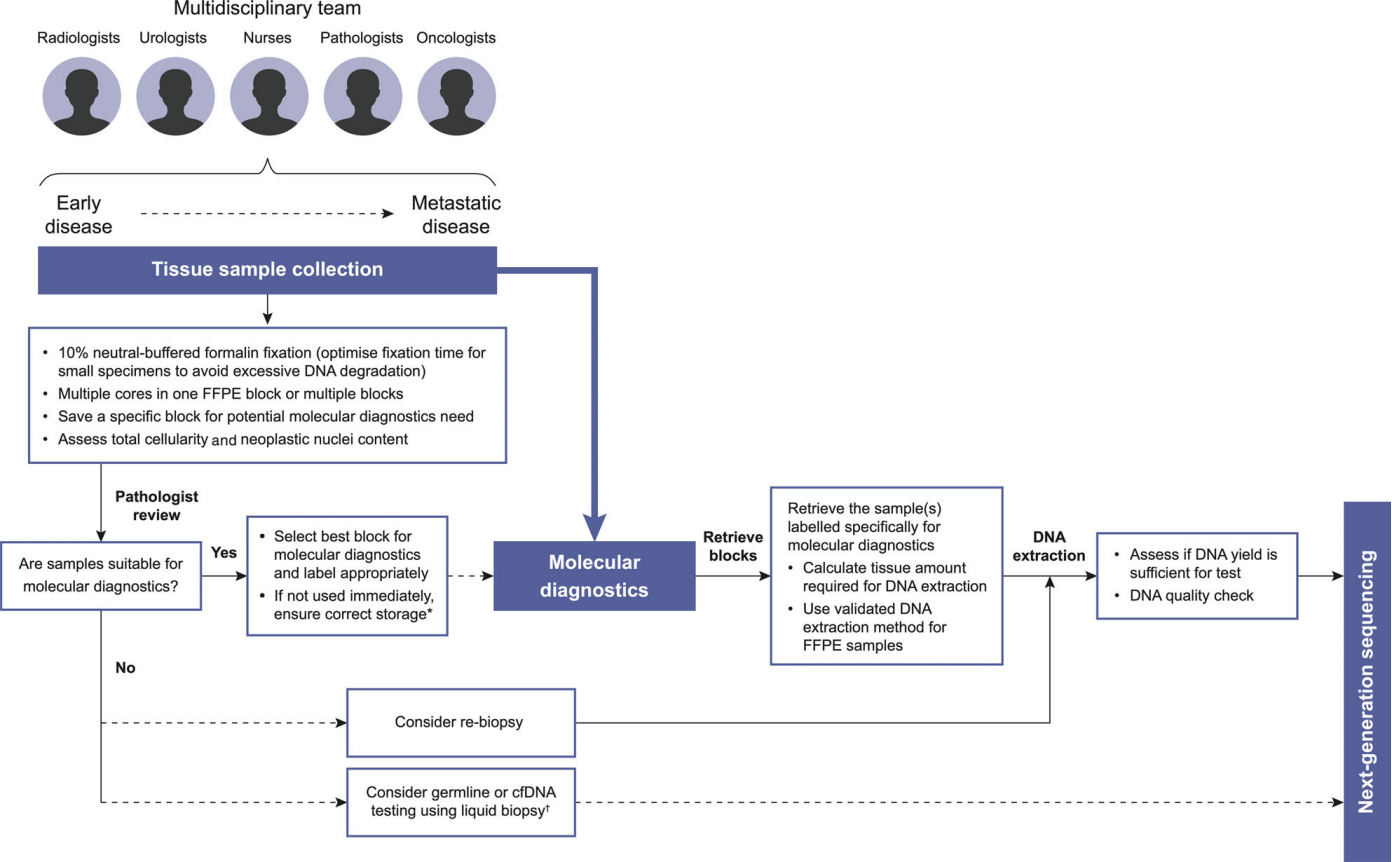
Schematic representation of tissue sample pathway for molecular diagnostic testing. cfDNA, circulating cell‐free DNA. *Storage conditions 18–25 °C and low humidity. ^†^For liquid biopsies, use cell‐stabilisation tubes and process within 3 days.

Pathologists, radiologists, and urologists have clear protocols for the collection of prostate tissue samples for diagnosis and Gleason scoring [43, 44], and guidelines for best practice in biospecimen collection and processing are available [45, 46, 47]. There are not yet, however, international standard protocols or specific guidance to obtain prostate tumour samples to aid the implementation of molecular diagnostic testing in routine clinical practice. Figure 2 provides a schematic representation of the tissue collection methodology, addressing the factors to be considered to improve testing success rates. Table 4 lists the factors and recommendations for formalin‐fixed and paraffin‐embedded (FFPE) sample collection, processing, and storage. During histopathological diagnosis and staging, the diagnostic pathologist should preserve and label a ‘molecular diagnostic’ FFPE block where haematoxylin and eosin (H&E) staining shows sufficient cellularity and tumour content for genomic analysis. For HRR alteration testing in mCRPC, suitable specimens should contain enough cellularity to yield the required DNA amount for the local test and a minimum neoplastic cell content (e.g. 10–30%, depending on the test used and local validation data and whether sequence variants only or copy number variants are being screened for) to ensure variants can be easily detected and distinguished from deamination or oxidation artefacts and other sequencing background noise [48]. Low tumour content not only impedes detection of low allele frequency somatic mutations but also affects the correct assessment of copy number variations as these may be diluted into the normal copy number profile of non‐tumour cells in the samples; this is particularly relevant to identify patients with intragenic or homozygous *BRCA2* deletions. Practical recommendations to assess cellularity and neoplastic content for different genomic applications are available online [49]. New, more accurate methods to obtain tumour tissue, such as targeted prostate biopsies using multi‐parametric magnetic resonance imaging, where available, can also help increase tumour content [50].

**Table 4:** Recommendations for processing and storage of FFPE samples for DNA analysis.

Factor	Recommendation
Tumour size	>5000 total nucleated cells with >10–20% neoplastic content*
Fixation method	10% buffered formalin
Fixation time	As short as possible (e.g. 3–6 h for core biopsies; maximum 24 h)
Knife blades	Replace before each block is cut to prevent cross‐contamination by tissue‐related nucleic acids
Number and thickness of sections	5–10 sections of 5–10 μm, depending on the dissected tissue size and cellularity
Decalcification procedure	Not recommended but if required for bone samples, use EDTA instead of acidic decalcification
FFPE block storage	Store in controlled environment (e.g. low humidity, 18–25 °C) to reduce oxidation and degradation of nucleic acids

EDTA, ethylenediaminetetraacetic acid.

* 5000 cells contain approximately 30 ng of DNA. At least 10–20% tumour content is required to reliably detect somatic variants at >5% allele frequency; higher tumour content may be required for detection of large somatic deletions and rearrangements.

## Factors affecting optimal sample type

**Figure 3:**
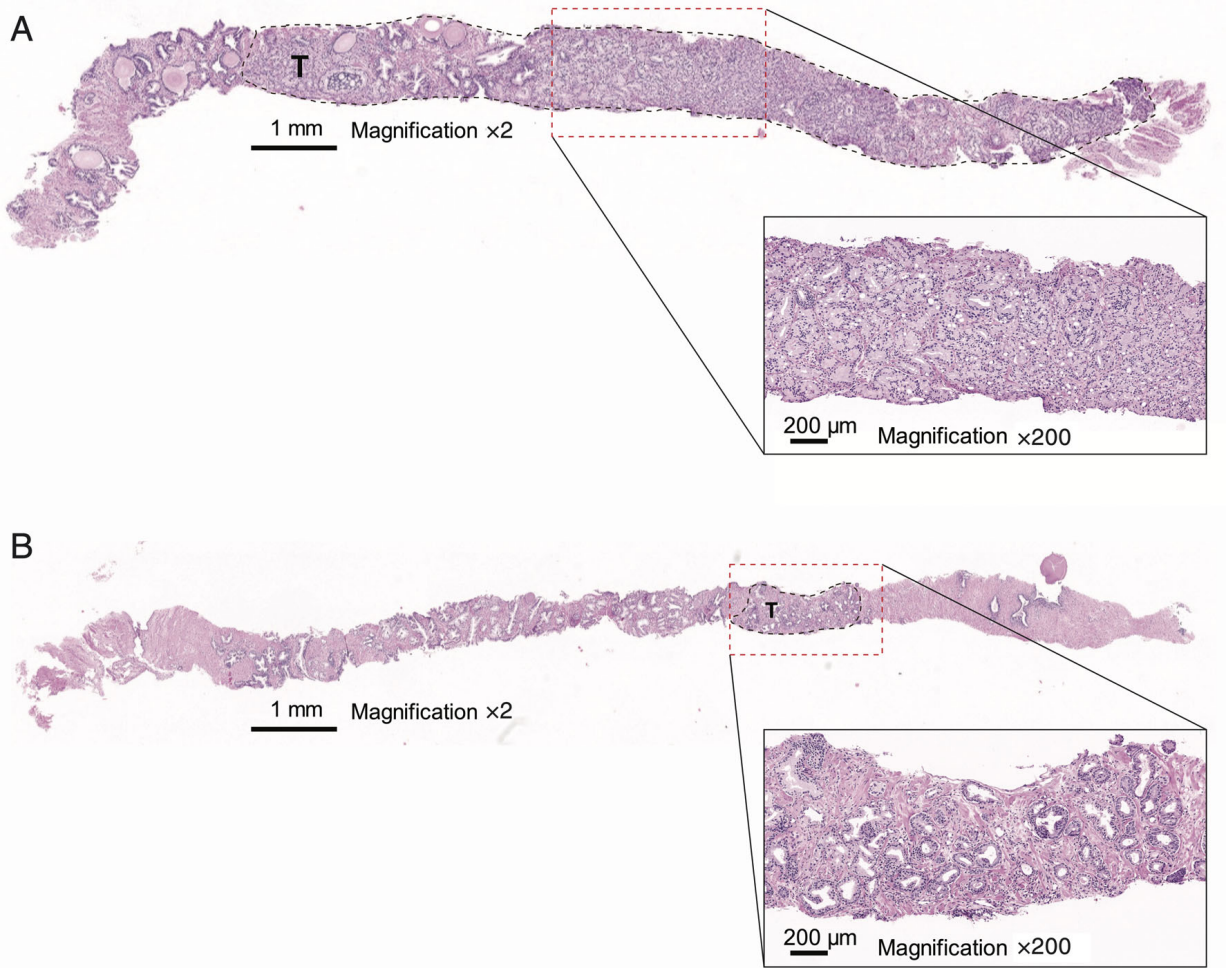
Illustrative examples of differences in neoplastic cell content in two prostate biopsy samples. (A) Core needle biopsy sample of approximately 1 mm × 10 mm showing that the sample predominantly has neoplastic cell content with minimal non‐neoplastic prostate cell content or adjacent soft tissues and (B) similar‐sized core needle biopsy showing much less neoplastic cell content and more non‐neoplastic content. The black dashed line areas (marked T) indicate the area of neoplastic cells. Red dashed lines represent the area of magnification of the tumour cell content. H&E staining.

Selection of the optimal sample type is dependent on factors such as size, age, collection method, and organ site. Tumour size may be critical in ensuring that the required quantity and quality of DNA are available for analysis, although this is highly dependent on cellularity. Surgical specimens, such as radical prostatectomies (available in approximately 10–15% of mCRPC patients), may provide a large amount of material, but this does not always translate to sufficient quantity/quality of DNA for testing if the tumour area is small and tumour cellularity is low. Conversely, smaller biopsy samples (i.e. core needle biopsy), typically used at initial diagnosis, may have limited tumour tissue for molecular testing after pathology diagnosis and grading, although they can provide good‐quality DNA as processing and fixation steps can be carefully controlled. For example, a small core needle biopsy of 1 mm × 10 mm may contain thousands of neoplastic cells with >80% tumour content and a yield >100 ng of DNA (such as shown in the example in Figure 3A), while another biopsy of similar size could be mostly non‐neoplastic cells, rendering it unsuitable for molecular analysis (such as in Figure 3B). Pooling of multiple cores from more than one biopsy may increase the yield of DNA, while macro‐dissection of the tumour area is recommended to increase the neoplastic content of the sample. Although there is a small risk that this practice may dilute the inter‐lesion heterogeneity of multifocal tumours, there is currently insufficient data regarding heterogeneity of HRR alterations in prostate cancer.

Sample age is also known to influence testing success; DNA extracted from newly collected FFPE samples is generally of adequate quality, although there is a gradual decline over time due to degradation and chemical modification. In the absence of newly collected FFPE samples, archived samples can provide successful test results, indicating that the preservation of DNA is achievable with optimisation of fixation and storage conditions. Findings from the PROfound study identified a decrease in test success rate with increased age of archived samples; however, successful tests were obtained in a proportion of samples that had been archived for >10 years [51].

Collection and processing of samples from metastatic biopsies are associated with challenges. Osteoblastic bone lesions are the most common metastases in patients with prostate cancer [52], and collection from this site presents issues for patients and the clinical team, including toxicity, invasiveness of the procedure, requirement for anaesthetic, and costs, such that clinicians may not pursue collection. Furthermore, processing of bone biopsy samples that require decalcification may lead to a reduction in the quantity and quality of DNA, and therefore, if required, EDTA must be used instead of harsher decalcification [53].

While there may be concern about whether a sample from an archived primary tumour is representative of distant metastatic disease at the time of consideration of PARP inhibitor treatment, evidence from the PROfound study showed that successful testing was undertaken with both primary and metastatic tumour samples, with the overall prevalence of HRR alterations being similar (27.2 and 31.8%, respectively) [18, 51]. Beyond germline mutations, findings from a small series of longitudinal samples from the same patient suggest that, at least for *BRCA1* and *BRCA2*, somatic HRR mutations are usually detectable in primary tumours in comparison with other genomic events, such as *AR* alterations, that emerge later in response to treatment‐selective pressure [17, 22]. Although there are challenges associated with sample collection and processing, clinical studies have shown that approximately 60–70% of primary and metastatic samples from patients with prostate cancer have successful test results [9, 17, 23, 24]. These findings highlight that the optimisation of diagnostic tissue collection and processing to provide an adequate quantity of high‐quality tumour samples is crucial for the testing process as primary specimens are currently the preferred source of material for HRR analysis [11, 18, 25, 51].

## Practical considerations and recommendations for prostate cancer tumour molecular diagnostic testing

Increased understanding of the link between molecular diagnostics and access to novel targeted therapies are likely to be significant motivating factors in implementing changes in the practice of tumour sample collection and processing. Involvement of the entire multidisciplinary team at the different stages of the patient's journey is critical to ensure that testing has a patient‐centric approach (Figure 2). Here, we provide a series of specific recommendations for different stages of the diagnostic pathway.

### Collection and handling of biopsy samples in pathology laboratories

Proactive identification of the most suitable sample for future molecular diagnostic testing should be championed by the diagnostic pathologist. Specific key recommendations for biopsy specimen handling are listed in Table 5. At diagnosis, adherence to pathology protocols can ensure rapid access to archived primary samples when the need for testing is identified and could significantly reduce the incidence of archived blocks being retrieved and found unsuitable for molecular diagnostics. The decision of whether to archive tissue samples as an FFPE block or extracted DNA may vary depending on the available facilities and institutional policies. Currently, long‐term storage of FFPE blocks is standard practice in many countries, including the European Union, Canada, and the USA, which are frequently archived at off‐site facilities, potentially leading to increased costs associated with sample retrieval and increased turnaround times. If no suitable sample is available, germline testing using blood samples or liquid biopsy with analysis of circulating cell‐free DNA (cfDNA) could be undertaken, or alternatively, re‐biopsy of a metastatic lesion could be considered.

**Table 5:** Specific key recommendations for biopsy specimen handling.

• Collect FFPE blocks specifically for molecular diagnostic testing (i.e. >20% tumour content and tumour cell rich) and clearly label them; this avoids tissue exhaustion and enables easy retrieval in the future
• Ensure pathologist is aware of potential future use of the specimens, particularly core needle biopsy, so they use minimal amount for histopathology, including immunohistochemistry; retain material from biopsies containing a significant amount of confirmed tumour material
• Embed multiple core needle biopsies in one FFPE block rather than pooling slides from different blocks to provide a DNA sample
• Consider DNA extraction from tissue samples at the time of diagnosis that can still provide sufficient quantity and quality of DNA; extracted DNA samples take minimal space in a freezer and, if appropriately extracted and stored, can last for decades without affecting quality.

### Processing specimens in molecular pathology laboratories

Guidance should be sought from appropriate laboratory technicians and scientists regarding the suitability of DNA samples for testing and DNA extraction procedures. Table 6 provides some specific key recommendations. Pre‐analytical quality control (QC) of DNA samples, including quantification of double‐stranded DNA yield and confirmation of the ability to amplify the DNA from sample or mean fragment size assessment, should be undertaken to minimise post‐library test failures [56, 57]. This should include evaluation of DNA amount (total), library QC, and quality of nucleic acids. Due to the need to sequence the entire coding regions of very large genes, NGS is the method currently used for HRR alteration testing. The panel of gene alterations to be evaluated should include *BRCA1* and *BRCA2* at a minimum, with other HRR genes being assessed depending on country‐specific approval. Evidence from breast and ovarian cancer studies has shown that an integrative NGS‐based approach is efficient to detect germline and somatic mutations in BRCA genes while simultaneously targeting a large spectrum of genetic alterations using FFPE tissue samples [58, 59, 60]. The chosen NGS approach should also be considered due to DNA requirements as some amplicon‐based NGS approaches (i.e. those using multiplexed primer pairs specific to the regions analysed to produce the required amplicons) only require approximately 10 ng of DNA, while targeted capture‐based NGS approaches (i.e. those using DNA or RNA probes to hybridise and capture the required genomic regions for downstream NGS) generally require more DNA (30–200 ng of DNA, depending on methodology used and local validation data) [61]. Ideally, laboratories performing capture‐based NGS approaches should aim for a minimum mean coverage of 500 unique reads (although less coverage is acceptable in cases with high tumour content), with at least 99% of coding regions being covered at >100×. For laboratories using amplicon‐based NGS approaches without de‐duplication strategies (e.g. unique molecular identifiers), local validation of required coverage is needed for different input DNA quantities and qualities.

**Table 6:** Specific key recommendations for specimen processing and analysis.

• Use validated DNA extraction protocol for FFPE that ensures appropriate quality and quantity of DNA for chosen methodology
• Perform pre‐analytical QC of DNA samples to minimise post‐library test failures
• Use a validated NGS assay (both amplicon‐ or capture‐based are potentially suitable) that should include entire coding regions for *BRCA1* and *BRCA2*, with other HRR genes being assessed depending on country‐specific licence indications and reimbursement approvals
• Perform QC of sequencing data generated according to laboratory policies and national and international guidelines [54, 55] to ensure appropriate level of coverage (see text) of all genomic regions reported by the assay

In addition to considering ways to improve tissue testing success rates, the time and cost consequences for test failures should be considered. A pathologist can identify samples likely to fail based on an existing H&E‐stained slide within minutes at a minimal cost, whereas retrieving and shipping a sample to a laboratory, annotation, macro‐dissection, DNA extraction, and QC checks take significantly more resource in terms of both time and cost. More importantly, a test failure, or the need to obtain a re‐biopsy, may mean a delay in a patient receiving the appropriate targeted treatment, which can be critical given the poor prognosis for patients with mCRPC. Overall, the turnaround time from receiving the sample in the laboratory to final report should be within 2–3 weeks. However, the time from request of the test to the sample being received in the laboratory can vary significantly and delay the whole process; this needs to be taken into consideration when designing efficient local sample pathways.

### Reporting tumour HRR alterations for treatment eligibility

Table 7 provides some key specific recommendations for reporting HRR alterations for treatment eligibility. In the mCRPC setting, only pathogenic or likely pathogenic mutations should be reported in the context of PARP inhibitor eligibility. Reporting of variants of uncertain significance (VUS) is not recommended for treatment eligibility, although some laboratory policies may require these to be included in the report. If VUS are reported, this must be reported separately to the main body of the report to avoid confusion and potential over‐treatment and unnecessary referrals to clinical genetics. The assignment of clinical relevance to findings using standardised scales, such as OncoKB Levels of Evidence scale or the ESMO Scale for Clinical Actionability of molecular Targets (ESCAT), can help to improve clinical interpretation of additional NGS findings and facilitate patient–physician discussion [62].

**Table 7:** Specific recommendations for tumour HRR alteration reporting.

• Only deleterious (pathogenic or likely pathogenic) mutations should be reported for PARP inhibitor eligibility
• If reported, VUS should be included separately from main treatment eligibility section of the report, and clearly state that no evidence is available suggesting a benefit for targeted therapies. These are not used for predictive germline testing
• Only mutations with variant allele frequencies above validated limit of detection of assay should be considered
• If the tumour assay is not capable of detecting larger chromosomal rearrangements, this should be clearly stated on the report; this will allow patients with strong family history to potentially be further investigated by germline testing

As tumour testing is routinely carried out using FFPE samples, there is a risk of artefacts of fixation/storage being considered bona fide mutations, particularly due to the deamination and oxidation of DNA. This problem can be ameliorated by using methods incorporating unique molecular identifiers or similar approaches. In addition, it is critical to only report variants found at variant allele frequencies higher than the validated limit of detection of the method used (approximately 5% when using FFPE) to avoid the reporting of false‐positive, artefactual results. A joint consensus recommendation for the interpretation and reporting of sequence variants in cancer compiled by the Association for Molecular Pathology, American Society of Clinical Oncology, and College of American Pathologists provides further details [54, 63].

### When should molecular testing be requested in the patient pathway?

Currently, among prostate cancer patients, only those with mCRPC are eligible for PARP inhibitor treatment, and so, molecular testing should be prioritised for these patients in routine clinical practice (Figure 2). Molecular testing of all men with newly diagnosed prostate cancer would currently involve a significant resource with very limited outcome in terms of targeted treatment as most patients with prostate cancer do not progress to metastatic disease. However, this situation may change in the future if targeted treatments became approved in earlier settings or if there is evidence that certain biomarker‐defined subgroups have a different prognosis, which may impact selection of the initial therapeutic approach. Given the potential delays in retrieving archival tissue, as well as the potential failure rates in up to 30–40% of specimens, consideration could also be given to retrieving diagnostic specimens for molecular testing at the time of metastatic disease and prior to progression to mCRPC, even though most patients will not progress on hormone therapy for 2–2.5 years. In addition, some centres may also consider HRR alteration testing in a wider patient population based on family history and/or aggressiveness of the tumour at diagnosis. Recent recommendations from ESMO also endorse academic centres and university hospitals in pursuing testing in wider populations, in the setting of clinical research programmes and after obtaining patient consent, in order to generate data to assess the value of testing in different disease settings that can help shape the optimal use of NGS testing in the near future and optimise the development of drugs currently in clinical trials [64].

### Informed consent and germline implications of tumour testing

The possibility of any deleterious or likely deleterious HRR alteration detected by tumour testing being of germline origin varies across populations but can potentially be more than 50% of all HRR gene alterations [42]. Many of the current guidelines advise that patients should be informed that tumour testing has the potential to uncover germline findings, which may warrant further investigation. NCCN guidelines recommend follow up for germline testing if tumour alterations, including *BRCA1* and *BRCA2*, are detected and/or if there is a strong family history of cancer [36], and ESMO guidelines recommend that patients with pathogenic mutations in cancer‐risk genes, identified through tumour testing, should be referred for germline testing and genetic counselling [41]. As the implications of a germline test result will have a significant impact not only on patients but also on their families, discussion of test results is highly recommended for patients who are referred for tissue testing. This may be undertaken by a urologist/oncologist before tissue testing or by medical geneticists after a relevant deleterious or likely deleterious HRR variant is identified on tissue testing.

## Alternative HRR alteration diagnostic tests

Tissue testing using FFPE specimens is currently the most widely used and standard approach for molecular diagnostic testing in most cancer types, including in mCRPC clinical trials [9, 27, 51]; however, there may be instances when this may not be an option. One alternative test that is under investigation uses a liquid biopsy or cfDNA [65]. Studies have shown that primary tissue and cfDNA share relevant somatic alterations, suggesting that cfDNA analysis may be a suitable surrogate for molecular subtyping in prostate cancer [66]. Some studies have included cfDNA assessments so that matched tissue and plasma samples, along with associated data on patient responses to treatment, can be compared to assess the relative benefits of both approaches [67, 68]. Genomic profiling of both cfDNA and FFPE tumour tissue samples using NGS from patients with mCRPC enrolled in the TRITON2 and TRITON3 studies successfully identified those with an HRR gene alteration for the evaluation of rucaparib [67]. Gene alterations in *BRCA1*, *BRCA2*, and *ATM* were detected in 2.0, 10.7, and 8.8%, respectively, of cfDNA samples and in 1.6, 8.2, and 5.8%, respectively, of tumour tissue samples [67]. Based on the findings of TRITON2 and PROfound, the FDA has approved the FoundationOne Liquid CDx test, a comprehensive pan‐tumour liquid biopsy test, for use as a companion diagnostic for rucaparib and olaparib, respectively [69].

Data from other studies in mCRPC are limited, although a retrospective study that evaluated gene alterations including HRR showed good concordance in BRCA alterations from cfDNA and FFPE tumour tissue samples [70]. Furthermore, good concordance in gene alterations between cfDNA and tumour tissue has been reported in other tumours such as non‐small cell lung and metastatic breast cancers [71, 72]. It is important to highlight that the gene alterations in cfDNA and FFPE samples can reflect germline alterations from normal cells as the DNA samples are derived from a combination of malignant and normal cells. In addition, there is a risk of clonal haematopoiesis of indeterminate potential (CHIP) interference in DNA repair genes. A recent study evaluating plasma cfDNA from 69 patients with advanced prostate cancer found that up to 10% of patients can have CHIP involving HRR genes (primarily *ATM* but also *BRCA2* and *CHEK2*), suggesting a need for paired whole‐blood samples as a control to avoid misdiagnosis [73]. Several guidelines and recommendations have been published for the handling and analysis of cfDNA samples in the clinical setting [74, 75, 76].

## Conclusions and future directions

Molecular diagnostic testing of patients with prostate cancer requires a multidisciplinary team approach in the era of precision medicine. As molecular profiling is a rapidly evolving field, education for pathologists and laboratory staff, in collaboration with radiologists, urologists, and oncologists, is needed for all aspects of collection, processing, storage, and availability of tumour tissue samples for molecular diagnostic testing, as well as an understanding of the NGS technology and diagnostic assays and the consequence of detection of germline variants for patients and families. The cancer geneticist/geneticist will be involved if the tumour testing suggests that there may be a germline mutation as this, if validated, could then involve testing family members. We recommend that considerations for molecular analysis be implemented in the diagnostic pathway of patients with prostate cancer to ensure that appropriate specimens are collected at diagnosis of metastatic disease and are suitable for genomic testing at the point of clinical decision‐making. With increased knowledge of the requirements for molecular profiling, greater adoption of best practices for genomic testing can be implemented both in local and reference centres. Optimisation of molecular diagnostic testing is not only feasible but also critical to ensure that patients with mCRPC, who would most likely benefit from targeted therapies such as PARP inhibitors, are identified.

## Disclaimer

This work includes contributions from, and was reviewed by, individuals who are employed by AstraZeneca and Merck Sharp & Dohme Corp., a subsidiary of Merck & Co., Inc.. The content is solely the responsibility of the authors and does not necessarily represent the official views of AstraZeneca or Merck Sharp & Dohme Corp., a subsidiary of Merck & Co., Inc.

## Author contributions statement

All authors contributed to the development and drafting of the manuscript and approved the final version for submission.
